# Navigating cholestasis: identifying inborn errors of bile acid metabolism for precision diagnosis

**DOI:** 10.3389/fped.2024.1385970

**Published:** 2024-04-05

**Authors:** Hiroshi Nittono, Mitsuyoshi Suzuki, Hiromi Suzuki, Satoru Sugimoto, Jun Mori, Rieko Sakamoto, Yugo Takaki, Hisamitsu Hayashi, Hajime Takei, Akihiko Kimura

**Affiliations:** ^1^ Division of Analysis Technology, Junshin Clinic Bile Acid Institute, Tokyo, Japan; ^2^ Department of Pediatrics, Juntendo University Faculty of Medicine, Tokyo, Japan; ^3^ Department of Legal Medicine, Showa University School of Medicine, Tokyo, Japan; ^4^ Department of Pediatrics, Graduate School of Medical Science, Kyoto Prefectural University of Medicine, Kyoto, Japan; ^5^ Division of Pediatric Endocrinology and Metabolism, Children’s Medical Center, Osaka City General Hospital, Osaka, Japan; ^6^ Department of Pediatrics and Pediatric Surgery, Juzen Hospital, Kumamoto, Japan; ^7^ Department of Pediatric Gastroenterology and Hepatology, Japanese Red Cross Kumamoto Hospital, Kumamoto, Japan; ^8^ Laboratory of Molecular Pharmacokinetics, Graduate School of Pharmaceutical Science, The University of Tokyo, Tokyo, Japan; ^9^ Department of Pediatrics, Kumamoto-Ashikita Medical Center for the Severity Disabled, Kumamoto, Japan

**Keywords:** inborn errors of bile acid metabolism, serum total bile acid, *γ*-glutamyltransferase, biliary atresia, open cholangiography

## Abstract

Inborn errors of bile acid metabolism (IEBAM) cause cholestasis during the neonatal period, and 8 types of IEBAM have been reported to date. IEBAM accounts for approximately 2% of cases of cholestasis of unknown cause. As only 10 patients have been identified in Japan, IEBAM presents diagnostic challenges due to the similarity of clinical symptoms with biliary atresia, thus necessitating precise differentiation to avoid unnecessary invasive procedures. Laboratory tests in IEBAM are characterized by normal *γ*-glutamyltransferase (GGT) and serum total bile acid (STBA) levels despite the presence of cholestasis; therefore, measuring STBA and GGT is essential to distinguishing biliary atresia from IEBAM. With suspected IEBAM, liquid chromatography–mass spectrometry (LC/MS) analysis of urinary bile acids is needed to optimize diagnostic and therapeutic efficacy and avoid open cholangiography and initiate treatment for primary bile acids such as cholic acid or chenodeoxycholic acid. This prospective report aims to increase awareness of IEBAM by highlighting the characteristics of general blood test and bile acid profiles from LC/MS analyses of blood, urine, and stool samples.

## 1 Introduction

Inborn errors of bile acid metabolism (IEBAM) is a disorder caused by the dysfunction of enzymes that mediate the cholic acid (CA) and chenodeoxycholic acid (CDCA) synthesis pathways. IEBAM is characterized by the accumulation of unusual bile acids or bile alcohols, which are intermediate metabolites in the bile acid synthesis pathway, in hepatocytes and the development of bile stasis–related liver damage (1). Currently, deficiencies in eight enzymes have been associated with IEBAM (Table 1), five of which have been reported in Japan: 3*β*-hydroxy-*Δ*^5^-C_27_-steroid dehydrogenase/isomerase (HSD3B7) deficiency (OMIM 607765), *Δ*^4^-3-oxosteroid 5*β*-reductase (SRD5B1) deficiency (OMIM 235555), 27-sterol hydroxylase (CYP27A1) deficiency (OMIM 213700), oxysterol 7*α*-hydroxylase (CYP7B1) deficiency (OMIM 603711), and bile acid conjugation defect–1 caused by a bile acid-CoA:amino acid N-acyltransferase (*BAAT*) gene mutation (bile acid conjugation defect–1: BACD1, MIM 619232) (2–5). In four of these conditions (except CYP27A1 deficiency), cholestatic liver injury develops between the neonatal and infancy periods, and it is thus essential to distinguish IEBAM from biliary atresia (BA), which requires surgical intervention.

**Table 1:** Eight types of inborn errors of bile acid metabolism.

Disease	Disease-specific bile salts and lipids	GGT	STBA	Gene
3*β*-Hydroxy *Δ*^5^-C_27_-steroid dehydrogenase/isomerase deficiency	G-*Δ*^5^-3β,7*α*,12α-triol-3S T-*Δ*^5^-3β,7α,12α-triol-3S	→	→	*HSD3B7*
*Δ*^4^-3-Oxosteroid 5β-reductase deficiency	GCA-*Δ*^4^-3-one TCA-*Δ*^4^-3-one	→	→	*SRD5B1*
Sterol 27-hydroxylase deficiency (cerebrotendinous xanthomatosis)	C_27__THC-3G Cholestanol	→	→	*CYP27A1*
Oxysterol 7α-hydroxylase deficiency	T-*Δ*^5^-3β-ol-3S	→	→	*CYP7B1*
Bile acid-CoA:amino acid *N*-acyltransferase deficiency	Unconjugated bile acids	→	↑	*BAAT*
Bile acid–CoA ligase deficiency	Unconjugated bile acids	→	↑	*SLC27A5*
α-Methylacyl CoA racemase deficiency	C_27__DHCA, C_27__THCA	↑	↑	*AMACR*
Cholesterol 7α-hydroxylase deficiency	LDL cholesterol, triglycerides	→	→	*CYP7A1*

GGT, *γ*-glutamyltransferase; STBA, serum total bile acid; G-*Δ*^5^-3β,7α,12α-triol-3S, glyco 3*β*,7α,12α-trihydroxy-5-cholenoic acid 3-sulfate; T-*Δ*^5^-3β,7α,12α-triol-3S, tauro 3*β*,7α,12α-trihydroxy-5-cholenoic acid 3-sulfate; GCA-*Δ*^4^-3-one, glyco 7α,12α-dihydroxy-3-oxo-4-cholenoic acid; TCA-*Δ*^4^-3-one, tauro 7α,12α-dihydroxy-3-oxo-4-cholenoic acid; C_27__THC-3G, 5β-cholestane-3α,7α,12α,25-tetrol 3-glucuronide; T-*Δ*^5^-3β-ol-3S, Tauro 3β-hydroxy-5-cholenoic acid 3-sulfate; C_27__DHCA, 3β,7α-dihydroxy-5β-cholestanoic acid; C_27__THCA, 3α,7α,12α-trihydroxy-5β-cholestanoic acid.

↑ Indicates elevated level, and → indicates within normal range.

Despite the presence of cholestasis, serum total bile acid (STBA) levels are normal or low in most cases, as measured using commercially available colorimetric assay kits, with the exception of amidation defects, and *γ*-glutamyltransferase (GGT) levels are also normal, except in *α-methylacyl* CoA racemase (AMACR) deficiency (OMIM 604489) (6). BA, by contrast, is the most common disease involving obstructive jaundice, with an incidence in Japan of approximately 1/10,000. The need for early detection of BA and early surgery has been noted (7). Because GGT and STBA levels are usually elevated in BA patients, the measurement of these two markers, along with determination of direct bilirubin level, should be performed to first rule out IEBAM before performing invasive inspections such as open cholangiography.

The Junshin Clinic Bile Acid Institute has performed urinary bile acid analyses in cholestasis of unknown cause since 1996 (8), with 10 cases of IEBAM noted (2–5, 9). In 2 of the 10 IEBAM cases (20%), open cholangiography, an invasive procedure, was performed to exclude BA (9, 10). This prospective report aims to increase awareness of IEBAM by highlighting the characteristics of general blood test and bile acid profiles from liquid chromatography–mass spectrometry (LC/MS) analyses of blood and urine samples.

## 2 Clinical course of two patients who received open cholangiography

### 2.1 Case 1 (HSD3B7 deficiency)

A 63-day-old Japanese infant with suspected BA was referred to the hospital by a general practitioner due to prolonged jaundice. Initial laboratory results showed elevated levels of alanine aminotransferase (ALT) at 735 U/L and direct bilirubin (D-Bil) at 5.5 mg/dl. In contrast, the STBA level was 6.7 μmol/L, and the GGT level was normal at 42 U/L. BA was ruled out at 74 days of age due to the passage of contrast medium through the intrahepatic duct and intestine on open cholangiography. Histological analysis revealed enlarged hepatocytes and the presence of bile plugs by hematoxylin-eosin staining. However, typical findings of BA, such as the proliferation of small bile ducts around the portal vein and fibrosis, were not observed. As the STBA levels were normal, we suspected IEBAM and analyzed urinary bile acid. Subsequent genetic analysis identified compound heterozygosity for c.805_811del:p.E269fs and c.839G>T mutations in the *HSD3B7* gene. This patient was identified as case no. 2. in reference (9). He is now 4 years old, growing and developing well, and his cholestasis resolved after oral administration of CA (10 mg/kg/day).

Analysis of bile acid in urine and serum was performed using LC-MS based on previously described methods (10–12). In Case 1, the analysis of urinary bile acids showed increased TBA, at 45.0 mmol/molC, excluding ursodeoxycholic acid (UDCA) (Table 2). By contrast, levels of the primary bile acids glycocholic acid (GCA) and taurocholic acid were extremely low, at 0.6 and 0.6 mmol/molCre, respectively. Additionally, levels of both glycochenodeoxycholic acid and taurocheno-deoxycholic acid were also extremely low, at 0 and 0.1 mmol/molCre, respectively, indicating defective primary bile acid production. On the other hand, the level of 3*β*-hydroxy-*Δ*^5^-bile acids was 41.2 mmol/molCre, which accounted for the majority (91.7%), excluding UDCA. Serum bile acid analyses showed a high content of 3*β*-hydroxy-*Δ*^5^-bile acids: 21.8 μmol/L (82.8%), respectively (Table 2).

**Table 2:** Urinary and serum bile acid analysis by LC/MS.

	Case 1				Case 2			
Sex (M/F)	M				M			
Country	Japan				Japan			
Diagnosis (reference no.)	HSD3B7 deficiency (9)				BAAT deficiency (BACD1) (10)			
First visit to the hospital (age, days)	63				23			
Initial symptoms	Prolonged jaundice				Prolonged jaundice, pale stool			
Bile acids analysis	Urine		Serum		Urine		Serum	
	mmol/molCr	(%)	μmol/L	(%)	mmol/molCr	(%)	μmol/L	(%)
Total bile acids	63.2		42.5		19.7		330.5	
(Excluding UDCAs)	45.0	(100)	26.3	(100)	19.6	(100)	329.9	(100)
Usual bile acids								
Conjugated primary bile acids	2.1	(4.6)	4.2	(16.0)	0.0	(0.0)	0.6	(0.2)
Non-conjugated primary bile acids	0.0	(0.0)	0.0	(0.0)	14.4	(73.5)	313.1	(94.9)
UDCAs	18.2		16.2		0.1		0.6	
Others	0.2	(0.4)	0.3	(1.0)	0.2	(1.0)	15.4	(4.6)
Polyhydroxylated bile acids	0.3	(0.7)	0.0	(0.1)	1.6	(8.2)	0.3	(0.1)
3β-Hydroxy-*Δ*^5^-bile acids	41.2	(91.7)	21.8	(82.8)	0.0	(0.0)	0.2	(0.1)
3-Oxo-*Δ*^4^-bile acids	0.0	(0.0)	0.0	(0.0)	2.6	(13.2)	0.0	(0.0)
Others	1.2	(2.6)	0.0	(0.1)	0.8	(4.1)	0.3	(0.1)

HSD3B7, 3β-hydroxy-*Δ*^5^-C_27_-steroid dehydrogenase/isomerase; BACD1, bile acid conjugation defect 1; UDCAs, ursodeoxycholic acid–related compounds; Polyhydroxylated bile acids, CA-1β-ol, CA-6α-ol, and CDCA-1β-ol.

Grey highlighting indicates disease-specific bile acids.

### 2.2 Case 2 (BACD1)

A 23-day-old Japanese infant, the second child of healthy parents, was referred to the hospital due to prolonged jaundice and pale stools. Initial laboratory results showed elevated ALT at 139 U/L, elevated D-Bil at 4.1 mg/dl, and elevated STBA at 147 μmol/L; however, the GGT level was normal, at 42 U/L. At 61 days of age, open cholangiography revealed the passage of contrast agent through the intrahepatic ducts and intestines, ruling out BA. Hematoxylin-eosin staining revealed enlarged hepatocytes and the presence of bile plugs, without fibrosis or loss of intrahepatic bile ducts. We initially suspected progressive familial intrahepatic cholestasis (PFIC), but the patient's parents did not show indication of pruritus during the child's 2-year follow-up, which we thought was not consistent with the clinical manifestations of PFIC. Subsequent genetic analysis identified compound heterozygosity for c.58C>T and c.953G>A mutations in the *BAAT* gene (10). At the time of the initial bile acids analysis, the patient did not have any symptoms of steatorrhea or fat-soluble vitamin deficiencies. The patient is now 5 years old and growing and developing well, with no cholestasis or elevated liver enzyme levels under oral administration of ursodeoxycholic acid (5 mg/kg/day).

In Case 2, the first bile acid analysis was performed 2.5 years after genetic testing led to a diagnosis of BACD1. The urinary TBA level was 19.7 mmol/molCre, with unconjugated primary bile acids accounting for the majority (73.5%) and no amino acid–conjugated bile acids present (Table 2). Trace amounts of sulfate-conjugated and glucuronide-conjugated bile acids were detected. The STBA level was significantly elevated, at 329.9 μmol/L with the majority (94.9%) being non-amino acid–conjugated primary bile acids, similar to urinary bile acids (Table 2). Thus, the urinary TBA level was low despite the abnormally high STBA levels.

## 3 Discussion

The four IEBAM disorders that can be treated via oral administration of unconjugated primary bile acids such as CA and CDCA are HSD3B1 deficiency (13), SRD5B1 deficiency (14), and CYP7B1 deficiency (15), which involve abnormal modifications of the cholanic acid backbone, and CYP27A1 deficiency (16), which involves abnormal side chain modifications. Generally, HSD3B7 deficiency is diagnosed based on an increased proportion of 3*β*-hydroxy-*Δ*^5^-bile acids; and SRD5B1 deficiency is diagnosed based on a high ratio of 3-oxo-*Δ*^4^-bile acids. Samples intended for analysis of these unusual bile acids must be stored in a cool place and analyzed rapidly by LC/MS due to their high susceptibility to oxidation (10). Bile acid amidation defects and bile acid–CoA ligase deficiency can be treated with GCA or UDCA (1, 17). A total of 12 types of IEBAM have been reported (2), including peroxisomal enzyme deficiencies such as Zellweger spectrum disorders (18), which respond poorly to oral primary bile acid administration. Approximately 2% of cases of cholestasis of unknown cause reportedly involve IEBAM (1), and some cases are likely missed. Of all types of IEBAM, HSD3B7 deficiency reportedly constitutes the highest number of cases (19–22), including one asymptomatic case reported in the 1930s (20). The authors also encountered a case diagnosed at the age of 22 years by urinary bile acid analysis (23).

IEBAM is characterized by STBA and GGT values that are low or within the normal range, despite elevated DB values seen in cholestasis (1). Bile acid analysis (e.g., by LC/MS) can be used to diagnose IEBAM due to increased levels of unusual bile acids. However, because enzymatic fluorescence is typically used for the analysis of STBAs in routine clinical practice, only bile acids with 3*α*OH groups can be measured (24), resulting in low or normal ranges. Caution should therefore be exercised when administering UDCA to patients with biliary stasis before a definitive diagnosis, as the 3*α*OH group is present and STBA levels are elevated. When IEBAM is suspected in a patient receiving UDCA treatment, STBAs should be measured after discontinuing UDCA for approximately one week. GGT is produced in the endoplasmic reticulum (microsomes) of the liver and bound to the bile canaliculus via glycosylphosphatidylinositol (GPI) anchors. When bile acids are present in excess in the bile canaliculus, the surfactant action of the bile acids disrupts GPI, allowing GGT to enter the sinusoids and increasing the blood concentration of GGT (25).

In Case 2, bile acid conjugation with taurine or glycine was defective, resulting in compensatory excretion of small amounts of sulfate-conjugated and glucuronide-conjugated bile acids into the urine. Under this condition, the blood GGT level is normal, because unconjugated bile acids are poor substrates of the bile salt export pump (BSEP) (26), an ABC transporter that localizes on the canalicular membrane of hepatocytes and mediates biliary excretion of bile acids. Similar to amino acid conjugation deficiency, PFIC types I and II are characterized by cholestasis with normal blood GGT levels (25). Both types of PFIC are caused by abnormal BSEP function. In PFIC type II, BSEP is down-regulated by a mutation in *ABCB11*, which encodes BSEP (27). In PFIC type I, BSEP is secondarily suppressed due to genetic deficiency in *ATP8B1* (28), which encodes FIC1, an enzyme that maintains phospholipid dynamics in the hepatocanalicular membrane (29) and/or regulates the FXR signaling pathway (30). Urinary bile acid analysis is practical for distinguishing amino acid conjugation deficiency from PFIC types I and II because it enables the evaluation of each bile acid, including unconjugated and conjugated forms.

**Figure 1:**
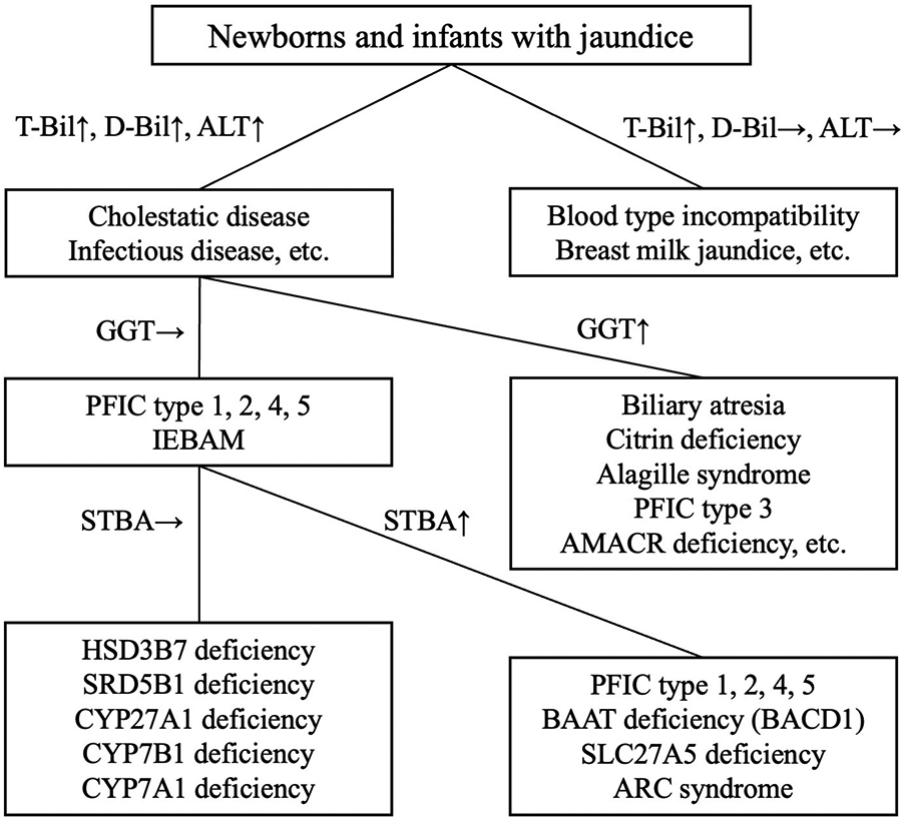
Flowchart for the diagnosis of IEBAM. ALT: alanine aminotransferase, T-Bil, total bilirubin; D-Bil, direct bilirubin; GGT, *γ*-glutamyltransferase; STBA, serum total bile acid; PFIC, progressive familial intrahepatic cholestasis; ARC, arthrogryposis-renal dysfunction-cholestasis; IEBAM, inborn errors of bile acid metabolism. See Table 1 for abbreviations of each IEBAM type. ↑ Indicates elevated level, and → indicates within normal range.

The diagnosis of BA is made through a comprehensive evaluation of laboratory data, including [1] blood biochemical tests showing the presence of cholestasis, in which elevated GGT and TBA levels can be seen; [2] ultrasonography of the porta hepatis to confirm the presence of a triangular cord sign; [3] bile sampling using a duodenal sonde to measure bilirubin; and [4] hepatobiliary scintigraphy. Based on the results of these tests, it may be necessary to confirm that no bile is flowing from the liver into the intestinal tract, and the Kasai procedure should be performed as early as possible (7). In IEBAM, STBA and GGT are generally in the normal range despite the presence of cholestasis; thus, measuring both of these parameters can help distinguish IEBAM from BA (Figure 1). Over the last 30 years, only 10 cases of IEBAM have been definitively diagnosed in Japan. Surprisingly, open cholangiography was performed in 2 of these 10 cases (20%), most likely due to a lack of knowledge regarding the IEBAM disease concept among general pediatricians and pediatric surgeons. The authors suggest that awareness of IEBAM should be increased in order to reduce the frequency of invasive and unnecessary laparotomies. Except for AMACR deficiency, most IEBAM patients have normal STBA and GGT levels in general blood tests. We highly recommend measuring STBA and GGT levels before oral administration of UDCA in cases of unknown cholestasis when seeing a patient with disease of unknown cause, followed by urinary bile acid analysis at least once.

## 4 Conclusion

Distinguishing IEBAM from other cholestatic disorders such as BA is crucial to ensure appropriate management. The limited number of patients with IEBAM in Japan underscores the need to increase awareness among healthcare professionals to avoid unnecessary procedures. Monitoring STBA and GGT levels, along with urinary bile acid analysis, can aid in accurate diagnosis and treatment planning, especially before initiating UDCA therapy in cases of unknown cholestasis.

## Data Availability

The original contributions presented in the study are included in the article/Supplementary Material, further inquiries can be directed to the corresponding author.
